# ATP13A2 Gene Silencing in Drosophila Affects Autophagic Degradation of A53T Mutant α-Synuclein

**DOI:** 10.3390/ijms24021775

**Published:** 2023-01-16

**Authors:** Nisha R. Dhanushkodi, Salema B. Abul Khair, Mustafa T. Ardah, M. Emdadul Haque

**Affiliations:** Department of Biochemistry and Molecular Biology, College of Medicine and Health Sciences, United Arab Emirates University, Al Ain P.O. Box 15551, United Arab Emirates

**Keywords:** α-synuclein, ATP13A2, autophagy, Drosophila, parkinsonism, sleep

## Abstract

Mutations in *ATP13A2* (*PARK9*), an autophagy-related protein, cause Kufor–Rakeb syndrome, an autosomal recessive, juvenile-onset form of parkinsonism. α-Synuclein (α-syn) is a presynaptic neuronal protein that forms toxic aggregates in Parkinson’s disease (PD). We studied α-syn aggregation and autophagic flux in ATP13A2-knockdown Drosophila expressing either wild-type (WT) or mutant α-syn. Dopaminergic (DA) neuron loss was studied by confocal microscopy. Sleep and circadian activity were evaluated in young and old flies using a Drosophila activity monitor. Thirty-day-old *ATP13A2*-RNAi A53T-α-syn flies had increased Triton-insoluble α-syn levels, compared to control A53T-α-syn flies without *ATP13A2*-RNAi. Whole-brain staining revealed significantly fewer dopaminergic (DA) neurons in the PPL2 cluster of 30-day-old *ATP13A2*-RNAi flies expressing WT-, A30P-, and A53T-α-syn than in that of controls. In *ATP13A2*-RNAi A53T-α-syn flies, autophagic flux was decreased, as indicated by increased accumulation of Ref(2)P, the Drosophila p62 homologue. *ATP13A2* silencing decreased total locomotor activity in young, and enhanced sleep features, similar to PD (decreasing bout length), in old flies expressing A53T-α-syn. *ATP13A2* silencing also altered the circadian locomotor activity of A30P- and A53T-α-syn flies. Thus, ATP13A2 may play a role in the autophagic degradation of A53T-α-syn.

## 1. Introduction

As a chronic neurodegenerative disorder, Parkinson’s disease (PD) affects 1% of the population older than 60 years of age [1]. It is characterized by classical motor symptoms, such as bradykinesia, rigidity, and resting tremor, as well as non-motor symptoms, such as sleep disturbances and depression [1,2]. Genetic risk factors can be identified in 5–10% of patients, and recent genome-wide association studies have shown the possible role of genetic factors in sporadic PD. PD pathology involves loss of dopaminergic (DA) neurons in the substantia nigra pars compacta (SNc), causing reduced facilitation of voluntary movements, as well as accumulation of α-synuclein (α-syn, a presynaptic neuronal protein) as intracytoplasmic eosinophilic inclusions in neurons (Lewy bodies and Lewy neurites) [3]. α-Syn appears to be central to pathogenesis of PD, as gene mutations, and duplications or triplication of α-syn locus are sufficient to cause PD [4,5,6].

Autophagy is a lysosome-mediated catabolic process in which dysfunctional organelles and misfolded proteins are degraded in mammalian cells. It plays pivotal roles in neuronal homeostasis [7]. Autophagy is among the major routes for the intracellular degradation of α-syn [8,9]. Accumulation of autophagic vacuoles and of LC3, a marker for autophagosomes, in the postmortem SNc of PD patients has been reported earlier [10,11].

Recessive mutations in several genes, including the ATPase type 13A2 gene (*ATP13A2*/*PARK9*), can cause neurodegeneration with a very young (juvenile) onset, usually presenting with other clinical signs in addition to parkinsonism (such as dystonia, oculomotor disturbances, pyramidal signs, and dementia). Parkinson disease type 9 (PD9) (also termed Kufor–Rakeb syndrome) is characterized by juvenile-onset, levodopa-responsive parkinsonism, pyramidal signs, dementia, and supranuclear gaze palsy. It is caused by recessive mutations in *ATP13A2* [12]. The gene encodes cation-transporting ATP13A2, an enzyme that is involved in the transport of divalent transition metal cations [13,14,15]. It appears to protect cells from Mn and Zn toxicity [15,16], possibly by causing cellular efflux and/or lysosomal sequestration of these metal ions [15,16]. However, it potentiates the toxic effects of Cd and Ni on developing neurites [13,17]. The exact function of ATP13A2 remains unclear, but it transports multiple cations (Mn, Ni, Cd, and Se) from the cytosol to the lysosomal lumen [18]. DA neuron loss caused by α-syn overexpression in animal and neuronal PD models is avoided by co-expression of ATP13A2. Moreover, the yeast ortholog of ATP13A2 helps to protect cells against Mn toxicity [19]. ATP13A2-deficiency causes Zn dyshomeostasis and mitochondrial dysfunction in hONs cells [15]. Zn dyshomeostasis caused by the loss of ATP13A2 leads to lysosomal dysfunction and α-syn accumulation in fibroblasts and mouse embryonic cortical neurons [14]. 

ATP13A2 also regulates lysosomal acidification, which is necessary for autophagosome–lysosome fusion and subsequent substrate degradation, and cells lacking ATP13A2 or fibroblasts of patients with ATP13A2 mutations displayed acidification defects [20]. In the frontal cortex of patients with PD, both increased and decreased ATP13A2 protein levels as compared to controls have been reported [21], whereas increased levels of ATP13A2 protein have been reported in the nigral dopaminergic and cortical pyramidal neurons of the PD patient’s brain [13]. 

P62 acts as an adaptor that binds ubiquitinated targets to autophagosome-associated lapidated microtubule-associated light chain 3 (LC3-II), leading to their engulfment by autophagosomes. P62 is strongly associated with intracellular clearance of misfolded proteins and defective organelles. It is a component of ubiquitin-positive inclusions found in various neurodegenerative disorders. In transgenic mice, p62-deficiency enhances α-syn pathology, in terms of the number of inclusions and staining intensity, as compared to mice that do express p62 [22].

In the present study, we examined the effect of ATP13A2 knockdown on the expression, aggregation, and neurotoxicity of wild-type (WT) α-syn, and A30P- and A53T-α-syn mutants in a Drosophila model. Recent findings in Drosophila and other PD model organisms enabled discovering new mechanisms and identified novel contributors to this disorder [23,24]. We silenced *ATP13A2* in Drosophila by using an RNAi construct, while expressing α-syn (WT/A30P/A53T) using GMR-Gal4 or Ddc-Gal4 drivers. The effects of *ATP13A2* silencing on α-syn expression and toxicity were studied by analyzing the α-syn protein levels, neurodegeneration, and Drosophila behavior, such as sleep. Our data suggested that the reduction in ATP13A2 contributed to increased aggregation of mutant A53T-α-syn with a concomitant increase in p62 levels, caused enhanced DA neuron loss, and exacerbated the non-motor phenotype in our experimental model.

## 2. Results

### 2.1. Targeted Silencing of Drosophila ATP13A2 Using RNAi

**Figure 1:**
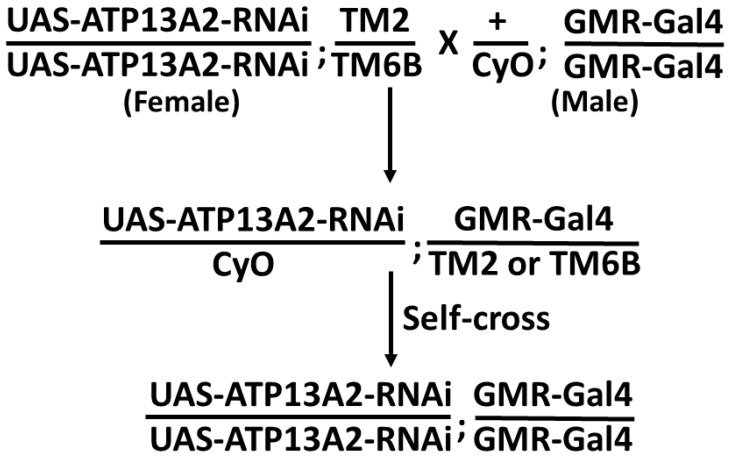
Schematic representation of the UAS-Gal4 system to generate flies silencing ATP13A2 under control of eye-specific driver GMR-Gal4. Virgin female flies are crossed with male flies to obtain the progeny. CyO, TM2 and TM6B are the balancer chromosome used to follow the target genes expression.

**Figure 2:**
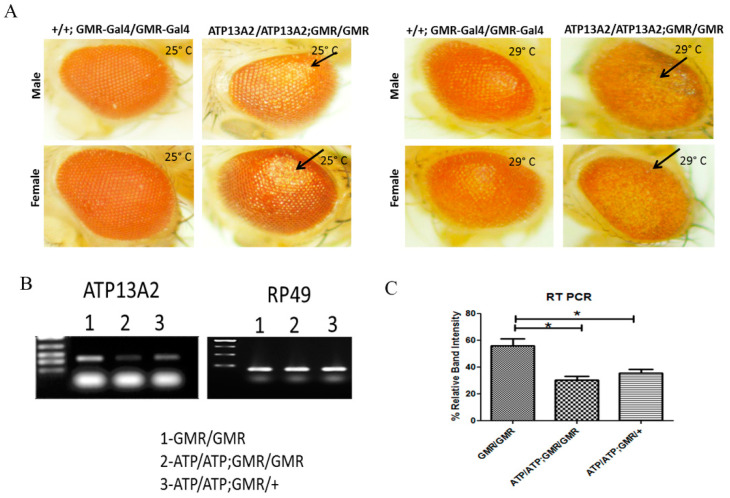
Targeted silencing of Drosophila ATP13A2 gene using RNAi (ATP13A2/ATP13A2). Defective eye phenotypes observed in male and female flies maintained at 25 and 29 °C carrying homologous copies of ATP13A2 RNAi with GMR-Gal4 as a driver when compared to eye phenotypes of flies with homologous copies of GMR-Gal4 alone. No gross phenotypic effect was observed in flies expressing a single copy of the GMR-Gal4 driver carrying ATP13A2 RNAi (**A**). RT-PCR confirmed significant silencing/downregulation of ATP13A2 in flies with homologous copies of ATP13A2 RNAi in the presence of GMR-GAL4 compared to the corresponding GMR-Gal4 control alone (**B**). Corresponding densitometric graph represents mean ± S. E. M for three independent experiments was shown (**C**). Differences were compared by Student’s *t*-test. * *p* < 0.05.

To study the role of ATP13A2 in PD, we used transgenic Drosophila expressing RNAi that silenced ATP13A2. These transgenic flies also co-expressed α-syn (WT/A30P/A53T) under UAS-Gal4 drivers, GMR-Gal4 and Ddc-Gal4. We have used the UAS-Gal4 bipartite system to generate transgenic flies silencing Drosophila ATP13A2 in the Drosophila eye to check the effect of ATP13A2 knockdown (Figure 1). GMR-Gal4 has been commonly used to express/downregulate the gene of interest in the developing eye to analyze the phenotypic effect of the gene. Examination of eye morphology revealed that flies with two copies of *ATP13A2*-RNAi exhibited an enhanced rough eye phenotype in the presence of the GMR-Gal4 driver, as compared to the control (GMR-Gal4 only) (Figure 2), at both 25 and 29 °C. 

To confirm *ATP13A2* knockdown in these flies further, semi-quantitative reverse transcription polymerase chain reaction (RT-PCR) was performed. This confirmed that there was significant downregulation of *ATP13A2* in tissue lysates of flies expressing two copies of *ATP13A2*-RNAi in the presence of the GMR-GAL4 driver, as compared to the corresponding GMR-Gal4 driver control flies (Figure 2).

### 2.2. Effect of ATP13A2 Silencing on Wild-Type and Mutatnt α-Syn Accumulation and Aggregation

**Figure 3:**
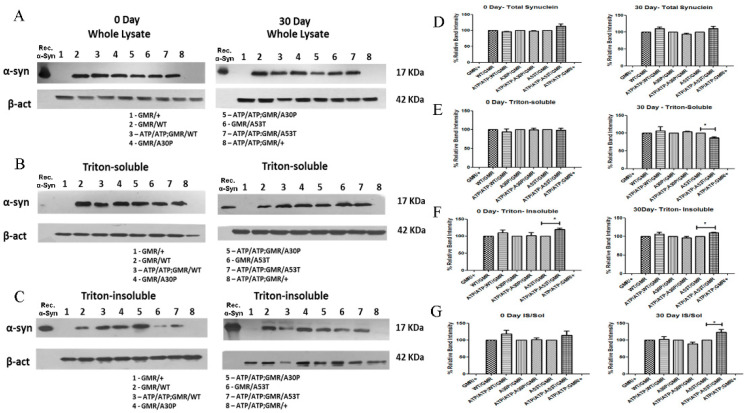
Effect of ATP13A2 silencing on α-syn protein expression and accumulation. Immunoblotting was performed using the whole-tissue lysis extraction method with fly heads (**A**), and densitometric analysis of the immunoblots revealed no accumulation of WT, A30P, or A53T α-syn during ATP13A2 silencing under GMR-Gal4 drive compared to control flies expressing α-syn alone (**D**). Triton-soluble α-syn levels in ATP13A2 RNAi flies expressing WT, A30P, and A53T syn were analyzed by Western blotting of day 0 and day 30 fly heads (**B**). The corresponding densitometric graph where ATP13A2 RNAi flies expressing WT, A30P, and A53T syn were normalized to 100% of the respective controls (GMR with α-syn) is shown (**E**). Triton-insoluble α-syn levels in ATP13A2 RNAi flies expressing WT, A30P, and A53T syn were analyzed by Western blotting of heads from day 0 and day 30 flies (**C**). The corresponding densitometric graph (**F**). Ratio of syn in the triton-insoluble to soluble fraction of Day 0 and Day 30 samples (**G**). The graph represents the mean ± S. E. M for three independent experiments. Differences in means were compared by one-way ANOVA followed by the Newman–Keuls Multiple Comparison post hoc test. * *p* < 0.05.

Next, we investigated whether *ATP13A2* silencing resulted in any alteration in α-syn protein expression levels when expressed together in flies. We found that there was no change in α-syn levels in whole lysates between the *ATP13A2*-silenced flies expressing WT or mutant α-syn and the corresponding control flies that expressed α-syn in the absence of *ATP13A2* downregulation. No increase in the level or accumulation of α-syn was observed both in 0-day-old and in 30-day-old flies. However, there was an increase in Triton-insoluble α-syn (which corresponds to α-syn aggregates) in *ATP13A2*-silenced A53T-α-syn-expressing flies, whereas no increased accumulation was noted in *ATP13A2*-silenced flies expressing WT-α-syn or mutant A30P-α-syn. We observed increased A53T-α-syn only when *ATP13A2* was downregulated in 0- and 30-day-old flies. Moreover, the increased ratio of insoluble to soluble α-syn was only observed in flies expressing A53T-α-syn and with silenced *ATP13A2*. Consistent with this change, in aged flies (30-day-old), we observed decreased levels of A53T-α-syn in the soluble fraction, suggesting that A53T-α-syn is more prone to aggregation (Figure 3). 

### 2.3. Neurodegeneration Is Increased in Flies Overexpressing α-Syn When ATP13A2 Is Downregulated

**Figure 4:**
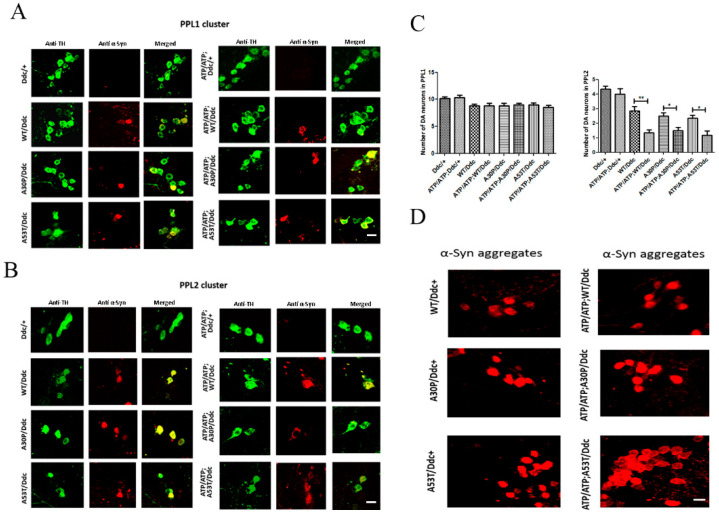
Neurodegeneration in ATP13A2 silencing flies expressing α-syn. Representative image of projected Z-series of PPL1 (**A**) and PPL2 (**B**) clusters in 30-day-old fly brains under the control of Ddc-Gal4 driver. The image shows co-staining of anti-TH (green) and anti-syn antibody (red) antibodies, revealing only a few neurons expressing α-syn in the PPL1 cluster compared to abundant expression in the PPL2 cluster in different genotypes under the Ddc-Gal4 driver. (**C**) Relative number of DA neurons within the PPL1 and PPL2 cluster of ATP13A2-silenced 30-day-old flies with Ddc-Gal4 driver-expressing WT, A30P, and A53T α-syn (n = 12) compared to age-matched control flies without ATP13A2 RNAi (n = 12). Differences in means were compared using one-way ANOVA followed by the Newman–Keuls Multiple Comparison post hoc test. ** *p* < 0.001; * *p* < 0.05. (**D**) Representative immunofluorescent staining of 30-day-old fly brain expressing WT, A30P, and A53T α-syn with or without ATP13A2 RNAi under Ddc-Gal4 driver stained for α-syn aggregates-specific antibody, showing a gross increase in aggregates in α-syn mutants, especially A53T, during ATP13A2 silencing. Scale bar 10 µm.

Next, we performed immunohistochemical analysis to examine whether silencing *ATP13A2* exacerbated α-syn toxicity in our flies. We chose to count the number of tyrosine hydroxylase (TH)-expressing neurons in the PPL1 and PPl2 clusters. TH is a rate-limiting enzyme for the biosynthesis of dopamine and highly express in dopaminergic neurons. We stained TH neuron clusters (PPL1 and PPL2) in *ATP13A2*-silenced fly brains under the control of the Ddc-Gal4 driver to examine the role of ATP13A2 on neurodegeneration. Ddc-Gal4 drives the transgene expression in TH-expressing neurons also known as dopaminergic neurons. We found that *ATP13A2* silencing increased neurodegeneration when α-syn was only overexpressed in the PPL2 cluster. Figure 4A,B show representative images of a projected Z-series of a 30-day-old fly brain under the control of the Ddc-Gal4 driver (with/without *ATP13A2*-RNAi) stained with anti-TH antibody, to label DA neurons. The relative number of DA neurons within the PPL1 and PPL2 clusters of 30-day-old *ATP13A2*-RNAi flies with Ddc-Gal4 UAS transgenes that expressed WT-, A30P-, and A53T-α-syn (n = 16), compared to age-matched corresponding control flies without *ATP13A2*-RNAi (n = 16), is shown in Figure 4A,B. Although we did not observe any degeneration of TH neurons in PPL1 clusters, there was a significant decrease in the number of TH neurons in flies expressing α-syn (WT/A30P/A53T) as compared to control Ddc/+ flies without α-syn expression. To confirm the expression of α-syn in TH neurons in our experimental flies under the Ddc-Gal4 driver, we co-stained TH neurons and α-syn. Figure 4A,B show representative images of the projected Z-series of PPL1 and PPL2 cluster in a 30-day-old fly brain with Ddc-Gal4 UAS transgenes co-stained with anti-TH (green) and anti-α-syn (red), confirming the expression of α-syn in DA neurons of different genotypes.

We sought to confirm our Western blot findings suggesting an increase in A53T-α- syn aggregation after ATP13A2 silencing. For this purpose, we used a conformation-specific antibody for α-syn, which stains only α-syn aggregates. Representative immunofluorescent staining, with a conformational antibody for α-syn aggregates, of 30-day-old fly brain expressing WT, A30P, and A53T α-syn (under control of the Ddc-Gal4 driver) with/without *ATP13A2* silencing, is shown in Figure 4D. We found a gross increase in the number of neurons stained for α-syn aggregates in *ATP13A2*-silenced fly brains expressing A53T-α-syn as compared to that in A53T-α-syn fly brains without *ATP13A2 silencing*. However, we were not able to quantify the α-syn aggregates in TH neurons since co-labelling with TH antibody did not work. Therefore, further studies are required to confirm this finding. 

### 2.4. Effect of ATP13A2 Silencing on the Autophagic Marker p62

**Figure 5:**
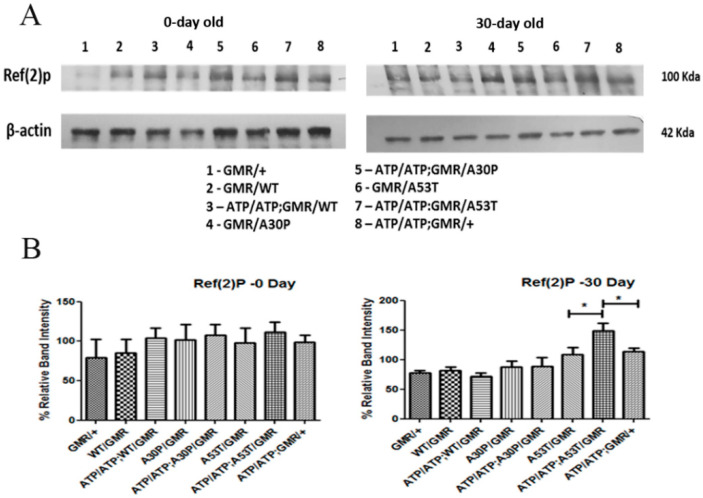
ATP13A2 silencing causes autophagy change in autophagic marker (p62) of mutant α-syn *A53T.* Expression of Ref(2)P, the Drosophila homologue of p62 in 0- and 30-day-old fly (**A**). Immunoblots showing an increase in Ref(2)P in ATP13A2-silenced flies expressing A53T compared to A53T alone, suggests inhibition of autophagy when ATP13A2 is silenced (**A**). Corresponding densitometric graphs (0- and 30-day-old fly) where ATP13A2 RNAi flies expressing WT, A30P and A53T α-syn is normalized to 100% of respective controls (GMR with α-syn) is shown (**B**). Graph represents mean ± S.E.M. for 3 independent experiments. Differences in means were compared by one-way ANOVA followed by the Newman–Keuls Multiple Comparison post hoc test. * *p* < 0.05.

The Drosophila orthologue for p62 is Ref(2)P. P62 has been known to mark proteins for autophagic degradation and is a regulator of protein aggregation in the adult brain. P62 interacts with polyubiquitinated protein aggregates through a ubiquitin-binding domain and with LC3 through its LC3-binding domain, thereby targeting these aggregates for degradation in autolysosomes. Ref(2)P has been shown to localize to age-induced protein aggregates as well as to aggregates caused by reduced autophagic or proteasomal activity. Bands were observed around 65 (expected) and 100 (observed) Kda. The high-molecular-weight 100 kDa protein was considered to represent P62 bound to high-molecular-weight protein aggregates. We used GMR-Gal4 driver to downregulate ATP13A2 and/or express α-syn. Fly heads were collected for Western blot analysis. We did not observe significantly increased levels of P62 in 0-day-old WT or mutant (A30P- and A53T-α-syn) flies with silenced *ATP13A2*, as compared to the corresponding controls. In 30-day-old flies, however, only ATP13A2-silenced A53T flies showed increased p62 as compared to the corresponding control A53T flies. Although very low expression of p62 was seen in GMR control young flies (0-day-old), we observed age-related accumulation of p62 in 30-day-old control flies. Control ATP13A2-silenced young flies (that did not express α-syn) also showed a slightly increased p62 level, as compared to control GMR young flies, but were almost the same at 30-day levels. ATP 13A2 silencing thus resulted in slight P62 accumulation at an early stage in control and flies that expressed WT or mutant α-syn. However, only flies expressing mutant α-syn A53T with ATP13A2 silencing showed a pronounced effect in old flies (Figure 5).

### 2.5. Effect of ATP13A2 Silencing on Sleep in Young and Old Flies

**Figure 6:**
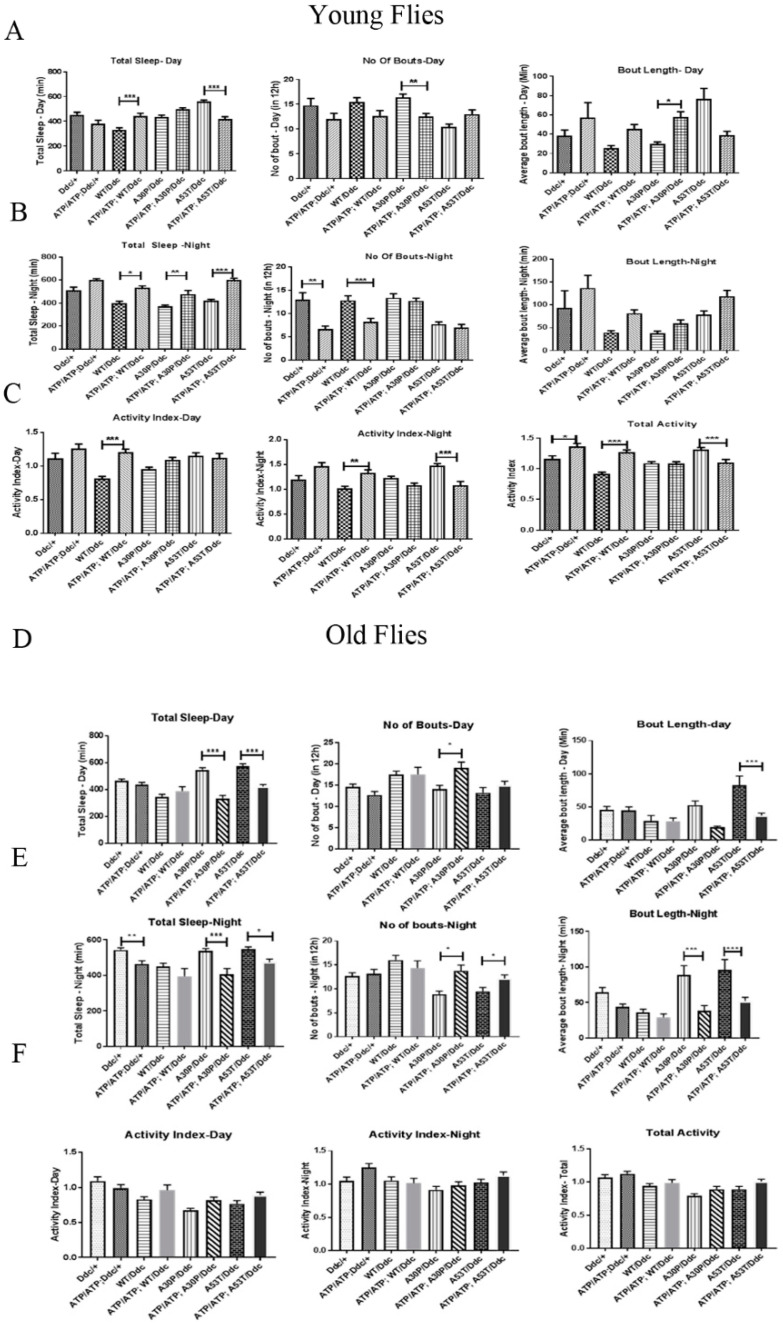
Decreased locomotor activity in ATP13A2 young flies expressing A53T mutant. Sleep analysis was performed in ATP13A2-silenced young flies (0–3 days after hatching) and old flies (30-day-old) expressing wild-type (WT), A30P and A53T mutant α-syn using Ddc-Gal4 driver. Total length of sleep, fragmentation in sleep indicated by sleep bout number and the average length of sleep indicated by sleep bout length in 12 h light (Day) and 12 h dark (Night) cycle was analyzed in young flies (**A**,**B**) and old flies (**D**,**E**)**.** The activity index represents fly activity level during the wake periods in day, night and in total for young and old flies (**C**,**F**). Total activity in young flies was decreased in A53T flies when ATP13A2 was silenced (**C**). In old flies, both mutants A30P and A53T showed a decreased night-time sleep with increased sleep fragmentation (**E**). Bars represent mean values of at least three independent experiments ± the standard error mean (S.E.M.). Each experiment was conducted with 16 flies that were individually recorded using drosophila activity monitor. Differences in means were compared by one-way ANOVA followed by the Newman–Keuls Multiple Comparison post hoc test. *** *p* < 0.0001, ** *p* < 0.001, and * *p* < 0.05.

All the α-syn-expressing young flies, when compared with ATP13A2 RNAi flies expressing α-syn under the control of the Ddc-Gal4 driver, showed pathogenic symptoms of α-syn night-time sleep (Figure 6). WT flies had decreased night-time sleep with an increased bout number and decreased bout length: A30P-α-syn-expressing flies had decreased bout length at day while A53T-expressing flies did not show any change in the number of bouts at night when compared with *ATP13A2*-silenced flies expressing A53T-α-syn. A53T-α-syn-expressing young flies showed increased daytime sleep with decreased bouts and increased bout length. In old flies, when ATP is silenced, A30P-and A53T syn-expressing flies show an increased number of bouts at night and decreased bout length at night. These flies also exhibited decreased sleep during the day and night (Figure 6). 

### 2.6. Effect of ATP13A2 Silencing on Circadian Locomotor Activity

**Figure 7:**
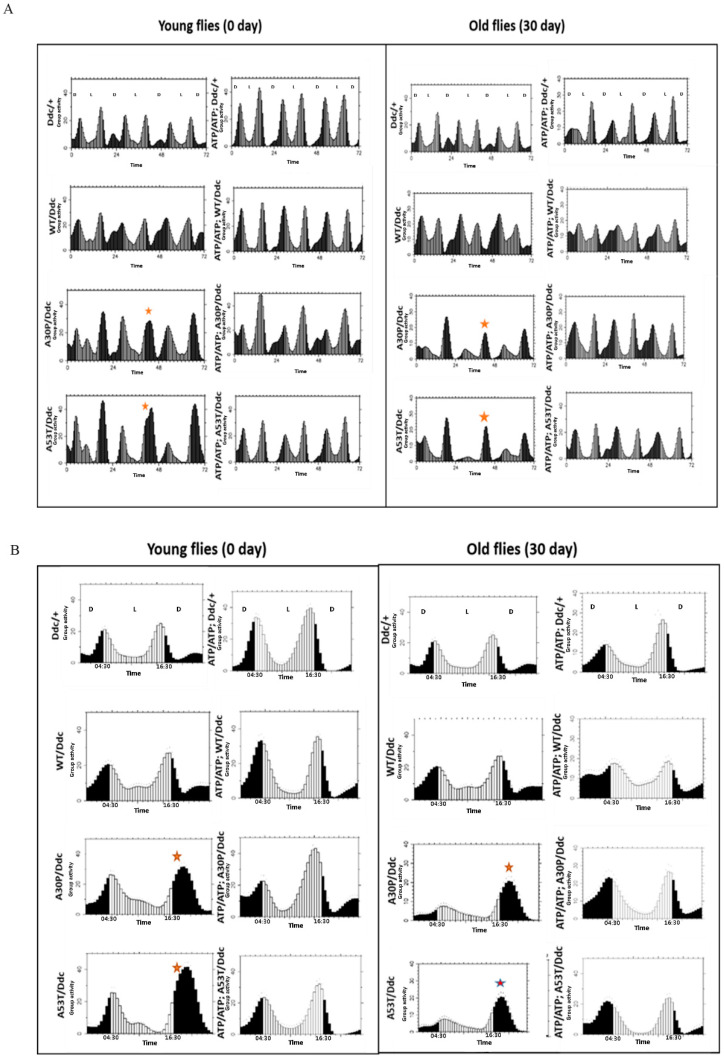
ATP13A2 silencing alters the circadian defect of flies expressing mutant α-syn. Locomotor activity and anticipation of the dark–light transition of young (0–3-day-old) and old (30-day-old) flies expressing WT, A30P, and A53Tα-syn with and without ATP13A2 silencing were studied using a Drosophila activity monitor (DAM) (**A**) and the corresponding Eduction graph is presented (**B**). Dark (12 h) and light (12 h) phases were indicated as D and L, respectively. Group activity (counts/min) was also shown. A30P and A53T flies (young and old) showed defective evening light expectancy, as indicated by * (**A**,**B**).

Dopaminergic neuronal pathways of Drosophila are involved in circadian rhythmicity. It has been documented that the expression of pre-fibrillar α-syn mutants in dopaminergic neurons affects circadian periodicity and locomotor activity in an age-dependent manner [25]. Thus, we have investigated whether the silencing of ATP13A2 in α-syn-expressing flies has any role in circadian behavior. We found that the mutant A30P and A53T α-syn-expressing flies in the presence of the Ddc-Gal4 driver showed disrupted circadian rhythmicity with regard to the evening activity peak. This defect was observed in both young and old flies (Figure 7; asterisk). It is noteworthy that this defect was absent when ATP13A2 was downregulated in these flies. It is not clear how ATP13A2 silencing altered such defect in A30P- and A53T-expressing files. Further studies are needed to confirm the role of ATP13A2 in circadian activity.

## 3. Discussion

Mutations in *ATP13A2* are associated with Kufor–Rakeb syndrome (KRS), an autosomal recessive form of juvenile-onset, atypical PD, known as PD-9 [11,26,27]. In the current study, we aimed to explore the effect of reduced ATP13A2 levels on the aggregation and toxicity of human WT or mutants α-syn (A30P- and A53T-α-syn) in a Drosophila model. We obtained flies with silenced *ATP13A2*, in the presence or absence of WT-, A30P-, and A53T-α-syn and studied the biochemistry of α-syn aggregation and its relevance to neurodegeneration, locomotor activity, and non-motor features, such as sleep behavior, in these flies. We confirmed the reduction in *ATP13A2* mRNA by semi-quantitative PCR. We also found that the reduction in ATP13A2 in the Drosophila eye caused severe eye defects, confirming the importance of ATP13A2 protein for proper maintenance of cellular integrity. 

It has been documented that point mutations, such as A30P in α-syn, have a tendency to aggregate more slowly than WT α-syn due to an impaired β-structure, while A53T-α-syn has been shown to form both soluble oligomers and insoluble fibrils more rapidly than WT α-syn [28]. We did not observe any change in the total α-syn levels in 0- or 30-day-old flies with *ATP13A2* silencing, irrespective of whether they expressed WT, A53T-, or A30P α-syn. However, upon processing the samples as Triton-soluble and -insoluble fractions, we observed that the level of A53T-α-syn in *ATP13A2*-RNAi flies showed a slight decrease in the triton-soluble fraction as compared to A53T-α-syn control flies, at 30 days. Additionally, the level of A53T-α-syn levels were increased in the Triton-insoluble fraction at days 0 and 30, suggesting higher aggregation of A53T-α-syn when *ATP13A2* was downregulated. Additionally, the ratio of insoluble to soluble α-syn in *ATP13A2*-downregulated A53T-α-syn-overexpressing flies showed a similar trend. Thus, Western blot analysis revealed that monomeric α-syn levels were lower, with a corresponding increase in α-syn aggregates in the lysates of flies with co-expressed *ATP13A2*-RNA, as compared to control A53T-α-syn flies. This finding was further confirmed by confocal staining using a conformational antibody for α-syn (anti-α-syn filament antibody), in which A53T-α-syn *ATP13A2*-RNAi flies showed more α-syn aggregates than did A53T-α-syn flies without *ATP13A2*-RNAi. Although Western blotting results using GMR drivers provided strong evidence confirming this finding, we performed confocal imaging for syn aggregates using the TH driver to show α-syn accumulation in TH neurons. However, we were not able to quantify the filament-specific synuclein in TH neurons since co-staining with TH antibody did not work. Therefore, a more detailed study is necessary to evaluate the presence of aggregated syn in different clusters and its co-localization with multiple α-syn antibodies as well as TH antibodies. Based on our current observations, we speculate that α-syn with the A53T mutation undergoes a physiological change when ATP13A2 levels are low, possibly due to the decreased autophagic flux, forming toxic oligomeric species or high-molecular-weight aggregates. Recently, it has been shown that ATP132 recruits HDAC6 to facilitate phagosome–lysosome fusion [29], and that this process is important for the proper clearance of defective organelles and aggregated proteins [26], thus supporting our current findings.

To determine whether the expression of human α-syn may contribute to increased toxicity, we performed immunostaining in these flies using an antibody targeting TH-expressing neurons in the fly brain. Confocal microscopic analysis revealed significant neurodegeneration in aged α-syn-expressing flies with wild-type or mutant α-syn in the PPL2 cluster. Notably, neurodegeneration was aggravated in ATP13A RNAi flies expressing WT/A30P/A53T α-syn compared to the respective control flies that expressed only WT/A30P/A53T α-syn. This result suggests that reduced ATP13A2 levels interfere with phagosome-lysosome fusion, which perturbs the clearance of toxic α-syn, resulting in enhanced DA neuronal loss. It has been proposed that α-syn clearance can take place in lysosomes, and because of impaired lysosomal activity resulting from ATP13A2 downregulation, there is a buildup of misfolded proteins, such as α-syn, further contributing to intracellular toxicity. 

Patients with PD are known to suffer from non-motor symptoms, such as sleep abnormalities, a year before the diagnosis of the disease [30,31]. Our study revealed that mutant A53T-α-syn expression in *ATP13A2*-silenced old flies caused a marked decline in their awake activity, when studied using a Drosophila activity monitor (DAM). This pathology could be correlated to an increase in α-syn aggregation in A53T-α-syn *ATP13A2*-RNAi flies, which leads to increased DA neuron loss. We also observed decreased locomotor activity when *ATP13A2* was silenced in the young flies. However, the expression of mutant A53T-α-syn when *ATP13A2* was silenced caused a marked decrease in locomotor activity in old flies.

In conclusion, the current study showed that mutant A53T-α-syn forms more triton insoluble aggregates and conformation-specific α-syn filament (as evidenced by both Western blotting and immunohistochemistry, respectively), possibly due to changes in metabolism during *ATP13A2* silencing, leading to increased DA neuron loss, and affecting sleeping patterns in these flies. Our results showed that the increase in α-syn aggregation and neurodegeneration in A53T-α-syn flies with reduced *ATP13A2* expression may be due to altered α-syn clearance through autophagy. Thus, this study demonstrated that reduced expression of *ATP13A2*, associated with PD, may contribute to increased aggregation of mutant A53T-α-syn, exacerbating the phenotype in a fly model of PD.

## 4. Materials and Methods

### 4.1. Fly Stock and Maintenance

The following fly stocks, used in this study, were obtained from the Bloomington Drosophila Stock Center (Bloomington, IN, USA): UAS-h[WT]αSyn (8146), UAS-h[A30P] αSyn (8147), UAS-h[A53T]αSyn (8148), GMR-Gal4 (8121), Ddc-Gal4 (7009), and Cyo;TM2/TM6b (3604). The transgenic flies for UAS-ATP13A2-RNAi (105477) were obtained from the Vienna Drosophila Resource Center (Vienna, Austria). For targeted tissue-specific transgene expression, we used the UAS/Gal4 system [32]. GMR-Gal4 or Ddc-Gal4 driver lines [33] were crossed with UAS-transgene flies to downregulate dATP13A2 (using RNAi) and/or to overexpress WT/A30P/A53T α-syn. Drosophila ATP13A2 RNAi flies are denoted as ATP or dATP. Flies were routinely maintained at 25 °C (unless specified) on cornmeal yeast agar medium, using a 12 h light and 12 h dark cycle. Male flies were used in all experiments except for RT-PCR.

### 4.2. Semi-Quantitative RT-PCR

Total RNA was isolated from female fly heads at 0 and 30 days using TRIzol (Life Technologies, Carlsbad, CA, USA), followed by cDNA synthesis using the GoSCRIPT reverse transcription system (Promega, Madison, WI, USA). PCR was performed using the following primers specific for Drosophila ATP13A2 (dATP), and RP49 in a Veriti 96-well thermal cycler (Applied Biosystems, Foster City, CA, USA):

dATP (forward: 5′-CAA CGC GAG CGA TAG TAC CT-3′

dATP reverse: 5′-GGA AGT GCA GGT GGG ACT AC-3′

RP49 forward: 5′-CCG CTT CAA GGG ACA GTA TC-3′

RP49 reverse: 5′TCT CCT TGC TTC TTG GAG GA-3′

PCR was conducted following the steps as mentioned below:

Initial denaturation at 95 °C for 1 min, and then 25 cycles starting with denaturation at 95 °C for 0.15 min, primer annealing at 61.5 °C for 0.15 min for ATP13A2 (for RP49, primer annealing temperature was 56 °C for 0.15 min), and extension at 72 °C for 0.30 min. In the last cycle, extension was continued at 72 °C for 7 min. The amplified PCR products were analyzed by electrophoresis on 1.5% w/v agarose gel containing ethidium bromide and visualized under UV illumination.

### 4.3. Immunoblotting

For whole-head lysates, male fly heads were homogenized by battery operated handheld “Micro-Vial Homogenizer” (Cat. BP-7005-000; Wilmad LabGlass, NJ, USA) in cell lysis buffer (5 µL/head) (Sigma–Aldrich, St Louis, MO, USA) containing protease phosphatase inhibitor and EDTA (1 mM). For each experimental group, 10–15 fly heads were collected and combined for protein samples preparation. The samples were then centrifuged at 6000 rpm (Eppendorf Centrifuge 5417R) for 20 min at 4 °C, and the supernatant was collected in fresh tubes. Samples were then mixed with 5× reducing sample loading dye and boiled for 5 min at 95 °C, cooled, briefly centrifuged, and then separated by sodium dodecyl sulfate–polyacrylamide gel electrophoresis (12%). For isolation of Triton-soluble and -insoluble fractions, we followed a previously described protocol [34]. Fly heads were homogenized in Triton lysis buffer (5 µL per head; 10–15 fly heads per group; 50 mM Tris-HCl, pH 7.4, 1% Triton X-100, 150 mM NaCl, and 1 mM EDTA, containing protease phosphatase inhibitor), centrifuged at 14,000 rpm (Eppendorf Centrifuge 5417R) for 20 min at 4 °C, and the supernatants were collected as Triton-soluble fractions. The pellets were suspended in 1× Laemmli buffer (2% SDS, 2% 2-Mercaptoethanol, 10% glycerol, 0.002% bromophenol blue, 100 mM Tris-HCl pH 6.8), heated at 95 °C for 10 min, and the supernatant was collected as the Triton-insoluble fraction. A BCA assay was carried out to determine the protein content in the Triton-soluble fraction before adding an equal volume of 2× Laemmli buffer. Extracts containing equal amounts of proteins (10–15 µg protein per lane) were electrophoresed on 4–12% gradient polyacrylamide gels (Bio-Rad, Hercules, CA, USA), and the proteins were transferred to a polyvinylidene fluoride membrane at 90 V for 90 min. The membranes were boiled in phosphate-buffered saline (PBS) for 5 min, blocked in 5% non-fat milk in PBS-Tween 20 (PBS-T; PBS plus 0.05% Tween-20), and probed overnight at 4 °C with a 1:3000 dilution of mouse monoclonal anti-α-syn (Syn-1) (BD Biosciences, San Jose, CA, USA). The membranes were washed several times with PBS-T and were incubated with a 1:80,000 dilution of horseradish peroxidase-conjugated goat anti-mouse antibody for 1 h at room temperature. After three washes in PBS-T for 30 min, the protein bands were visualized using a Super Signal West Femto Chemiluminescent Substrate Kit (Pierce, Rockford, IL, USA). Protein bands with correct size were detected and identified using prestained protein standards (Cat. Number 1610375, Bio-Rad, USA). Band intensities were quantified using Image J (NIH; Bethesda, MD, USA) software. The values are represented as percentages. The results are presented as the mean ± S.E.M of three independent experiments.

### 4.4. Sleep and Locomotor Activity Assays

Flies were maintained in a light:dark cycle (12 h:12 h) at 25 °C with equal population densities. Locomotor activity and sleep behavior were recorded from single males using the DAM system (Trikinetics, Waltham, MA, USA), as previously described [35,36]. The DAM monitor contains 32 channels, each connected to a small glass tube containing each fly, in which the movement of individual flies can be monitored as they break the infrared beam that bisects the tube. Movements were recorded in a 1-min bin size using the DAM 308 software. Sleep in Drosophila is defined as a bout of 5 or more minutes of inactivity [37]. The average duration of a sleep bout was calculated as the total amount of sleep (min) divided by the number of sleep bouts during the day and night. The activity index refers to the activity when flies were awake (the number of recorded movements divided by the total time [min]). Single males were recorded individually (16 flies per genotype) using the DAM system, and three independent experiments were performed for each genotype. With data extracted using DAM filescan110, sleep behavior was analyzed using Microsoft Excel.

### 4.5. Immunohistochemistry and Confocal Microscopy

Brains of different groups of 30-day-old flies were dissected in cold PBS and fixed in 4% paraformaldehyde in PBS for 1 h. The samples were washed and permeabilized overnight in 0.3% Triton X-100 in PBS (Wash buffer). Fixed brains were stained with mouse anti-TH antibody (mouse monoclonal, Immunostar)/anti-α-syn antibody (Syn1- Rabbit polyclonal, BD Biosciences)/anti-α-syn filament antibody (rabbit monoclonal, cat number ab209538; Abcam) at a 1:200 dilution for 48 h. After incubation, the brains were washed three times and incubated with the corresponding secondary antibodies overnight (anti-mouse Alexa 488/anti-rabbit Alexa 594) at 1:1000 dilution. After thorough washing, the stained brains were mounted using Fluoromount mounting medium (Sigma), and images were acquired using a confocal microscope (Nikon EZC1). DA neuron clusters were analyzed as previously described [38]. Optical sections of the brains were acquired at 40-μm intervals using a 40× objective for whole-brain imaging. Confocal stacks were merged into a single plane using Nikon EZC1 software (Nikon, Tokyo, Japan). The number of TH-positive neurons within the PPL1 and PPL2 DA neuronal clusters was counted by visual inspection of the individual confocal Z-series of images. An average of eight brains were analyzed in both hemispheres, for each genotype, and the results are expressed as the mean ± S.E.M.

## Data Availability

The authors declare that the data supporting the findings of the current study are available in the manuscript.
